# Draft Genome Sequence of Vibrio cyclitrophicus NCT10V, Cultivated from the Microbiome of a Marine Copepod

**DOI:** 10.1128/MRA.01208-19

**Published:** 2019-10-31

**Authors:** Ryan Nuttall, Gaurav Sharma, Pia H. Moisander

**Affiliations:** a Department of Biology, University of Massachusetts Dartmouth, North Dartmouth, Massachusetts, USA; b Institute of Bioinformatics and Applied Biotechnology, Electronic City, Bengaluru, Karnataka, India; Indiana University, Bloomington

## Abstract

Here, we report the draft genome sequence of Vibrio cyclitrophicus NCT10V, cultivated from the copepod Acartia tonsa, collected from coastal surface waters of the western North Atlantic Ocean. The assembly is 5,638,575 bp long and has 44 contigs, a GC content of 43.2%, and 5,044 protein coding sequences.

## ANNOUNCEMENT

Vibrio spp. are Gram-negative, curved, rod-shaped, motile cells (1) that often associate with marine invertebrates and particles, and some strains are linked with pathogenic and zoonotic diseases (2–5). Vibrio cyclitrophicus NCT10V was cultivated from the microbiome of a copepod (Acartia tonsa) collected from the coastal surface waters of the temperate western North Atlantic Ocean in January of 2016 (41.57 N, –70.80 W). Individuals were spread on nitrate-chitin-agar plates that were then incubated anaerobically in the dark at 25°C. Colonies were streaked for purification several times, transferred into marine broth (BD Difco, Franklin Lakes, NJ), and grown aerobically at 25°C with shaking. Genomic DNA was extracted using the DNeasy plant minikit (Qiagen, Valencia, CA, USA). Whole-genome sequencing was performed by Omega Bioservices (Norcross, GA, USA). For preparation of the whole-genome library, DNA was fragmented using a Bioruptor sonicator (Diagenode, Denville, NJ, USA), followed by a HyperPrep kit (Kapa Biosystems, Wilmington, MA, USA). Sequencing on an Illumina HiSeq 2500 platform with a paired-end 100-bp-run format resulted in 4,809,812 reads. Trimmomatic v0.35 was used with default parameters to remove the adaptor sequences from both ends and to filter the reads to retain sequences with a Phred score above 30 (6). Filtered and trimmed reads (3,772,769 reads; 78.5% of total) were assembled with SPAdes v3.9.1 (7) with default settings. The final assembly constituted a draft genome of 5,638,575 bp in 44 contigs, with a GC content of 43.2%. Contigs were cross-checked for any putative contamination via the Kraken-based taxonomic sequence classification system (8). The smallest and largest contigs were 587 bp and 746,755 bp, respectively, whereas the *N*_50_ value was 251,991 bp. RAST annotation (9) indicated 5,044 protein coding sequences, 62 tRNA genes, 7 rRNA genes (five 5S and one 16S and 23S rRNA each), and 34 repeat regions. Out of 5,044 proteins, 1,723 (34%) of the genes were assigned to SEED subsystems, whereas 3,474 (69%) were predicted to have a known (nonhypothetical) function. Several putative subsystems identified may be symptomatic of survival in a copepod microbiome, including nitrogen and phosphorus metabolism, virulence and defense, stress responses, motility and chemotaxis, and iron acquisition. These subsystems may aid in the acquisition of nutrients released by the copepod, alleviate the stress caused by living in association with a motile animal, and increase fitness under competition.

The 16S rRNA gene of NCT10V had the closest identity with *V. cyclitrophicus* ECSMB14105 (99.8% DNA identity; NCBI BLASTn), which was also the closest match (99.7% identity) based on the *toxR* gene, commonly used in *Vibrio* taxonomy (10). DNA-DNA hybridization (DDH) (11) values were 90.1 to 92.4 and 19.6 to 34.2 with *V. cyclitrophicus* and other select *Vibrio* spp., respectively (Table 1). Average nucleotide identity (ANI) (12) with genomes of other *V. cyclitrophicus* strains was 99.1 to 99.2% and substantially lower with other *Vibrio* spp. (Table 1).

**TABLE 1:** Summary of comparison of *Vibrio* spp. to *V. cyclitrophicus* NCT10V using ANI (12) and DDH (11)^*a*^

Organism and strain	ANI (%)	DDH (%)	GenBank accession no.	Isolation source^*b*^
*V. cyclitrophicus* FF160	99.2	92.2	GCA_000256605.2	Seawater (<1 μm)
*V. cyclitrophicus* 1F111	99.1	90.1	GCA_000247005.2	Seawater (1–5 μm)
*V. cyclitrophicus* 1F289	99.1	90.8	GCA_000256185.2	Seawater (1–5 μm)
*V. cyclitrophicus* ZF14	99.2	91.9	GCA_000256265.2	Seawater (>63 μm)
*V. cyclitrophicus* ZF28	99.2	92.3	GCA_000256285.2	Seawater (>63 μm)
*V. cyclitrophicus* ECSMB14105	99.2	92.4	GCA_005144905.1	Marine biofilm
V. alginolyticus ZJ-T	79.4	20.7	GCA_001679745.1	Epinephelus coioides
V. cholerae Env-390	77.5	19.6	GCA_001854425.1	Environmental
V. crassostreae 9CS106	88.1	34.2	GCA_000272185.2	Crab stomach contents
V. harveyi ATCC 43516	79.7	21.2	GCA_001558435.2	Shark
V. parahaemolyticus VP232	79.4	21.2	GCA_000454185.1	Human stool
V. vulnificus 101/4	78.7	19.9	GCA_000743155.1	Tilapia

a DDH was calculated using formula 2 (20).

b Values in parentheses indicate seawater size fraction.

AntiSMASH 5.0 (13) identified potential pathways for the secondary metabolites ectoine, cupriachelin, polyunsaturated fatty acid eicosapentaenoic acid, aryl polyene, beta-lactone, and bacteriocin. NCT10V has the *nrf* gene cluster (14, 15); thus, it can potentially complete dissimilatory nitrate reduction to ammonium (DNRA). A symbiotic polysaccharide (*syp*) gene cluster including the downstream regulator gene *sypG*, a mannose-sensitive hemagglutinin (MSHA) gene cluster, and several chitinase-encoding genes were identified, indicating that NCT10V is a member of the “L-strain” lineage that commonly associates with marine animal hosts and large particles and forms biofilms (16–19). The NCT10V genome could give insight into the evolution of the *Vibrio* genus and could facilitate future work on *Vibrio*-animal associations and DNRA.

### Data availability.

This whole-genome shotgun project has been deposited at DDBJ/ENA/GenBank under the accession number VUKB00000000. The version described in this paper is version VUKB01000000. The data are available under BioProject accession number PRJNA563747 and BioSample accession number SAMN12687761. The reads are available under SRA accession number SRP220210.
